# An atypical airway cast

**DOI:** 10.1093/icvts/ivad206

**Published:** 2023-12-14

**Authors:** Wincy Wing-Sze Ng, Max Kwun-Hung Wong

**Affiliations:** Adult Intensive Care Unit, Queen Mary Hospital, 102 Pok Fu Lam Road, Hong Kong Special Administrative Region, China; Department of Cardiothoracic Surgery, Queen Mary Hospital, 102 Pok Fu Lam Road, Hong Kong Special Administrative Region, China

**Keywords:** Inhalation injury, VV-ECMO, airway cast

## Abstract

A 60-year-old man intubated for airway protection after smoke inhalation was found to have decompensated hypercapnic respiratory failure. Fiberoptic bronchoscopy revealed obstructive airway slough and pseudomembrane, a manifestation of severe inhalation injury. Veno-venous extracorporeal membrane oxygenation was established for stabilization. The airway casts were removed successfully with periprocedural veno-venous extracorporeal membrane oxygenation support.

## DESCRIPTION

A 60-year-old man with unremarkable past health presented to the emergency department following smoke inhalation at a fire scene. The patient was unconscious with an initial serum carboxyhaemoglobin level of 44%. Emergency intubation was then performed. One hundred percent oxygen was administered until the carboxyhaemoglobin level normalized. Bronchoscopy on day 1 revealed soot deposition and scant sputum in the proximal airways. The patient required low ventilatory support with normal airway pressures. Bronchoscopy was repeated on day 2 mainly for upper airway assessment, which revealed significant laryngeal oedema, for which the patient was kept intubated.

**Figure 1:**
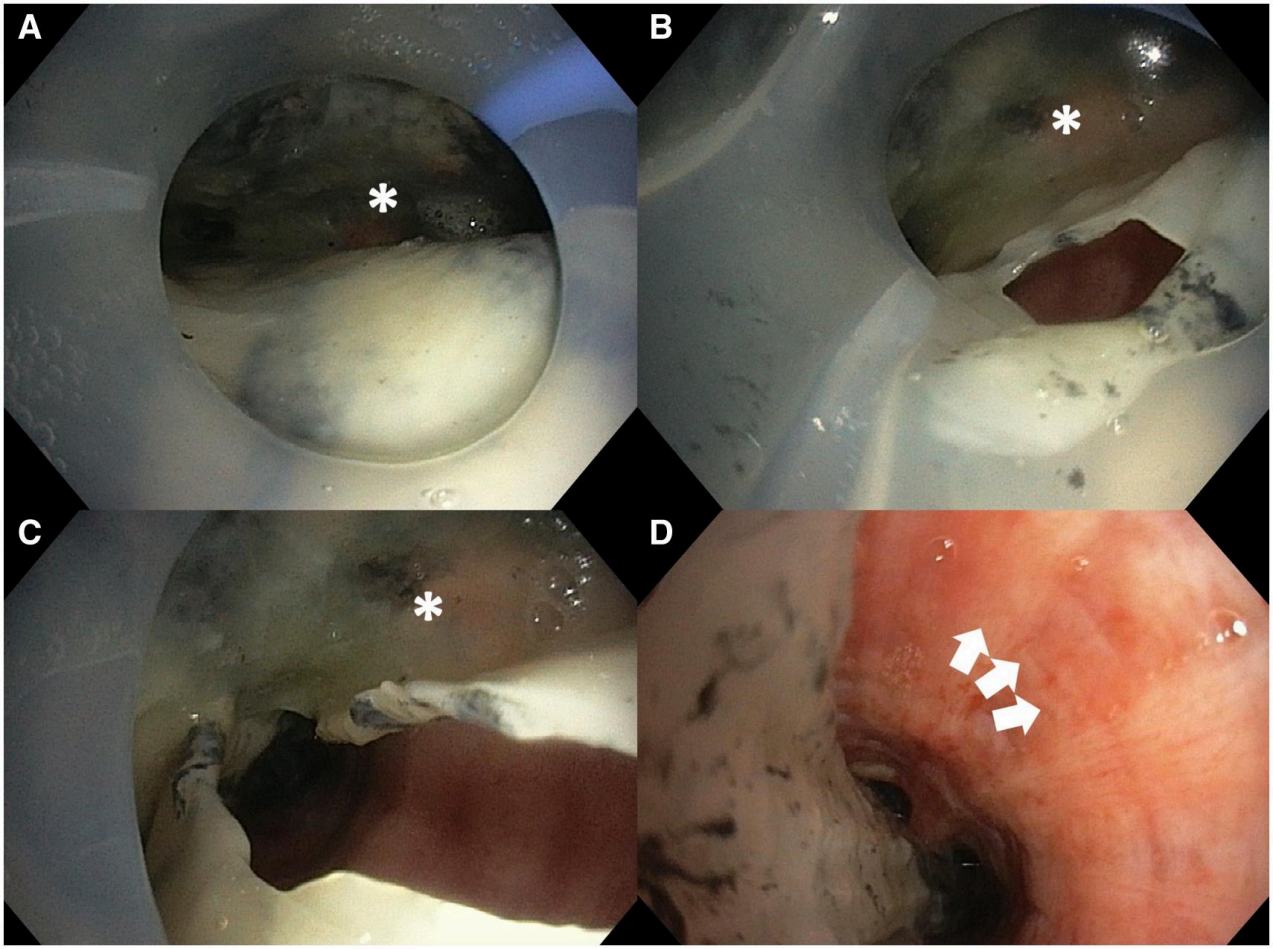
(**A**) Fiberoptic bronchoscopy image obtained at the time of elevated airway pressures. Extensive mucosal slough was seen distal to the tip of the endotracheal tube, causing mechanical obstruction and difficulty in ventilation. The asterisk (*) denotes the location of the carina. (**B**) The mucosal slough tissue was cut open with biopsy forceps bronchoscopically, revealing underlying inflamed mucosa. The asterisk (*) denotes the location of the carina. (**C**) Repeated attempts to remove the obstructive pseudomembranous slough tissue at the bedside was unsuccessful. The asterisk (*) denotes the location of the carina. (**D**) Intra-operative photo showing underlying inflamed mucosa (white arrows) after removal of the pseudomembranous slough tissue.

**Figure 2:**
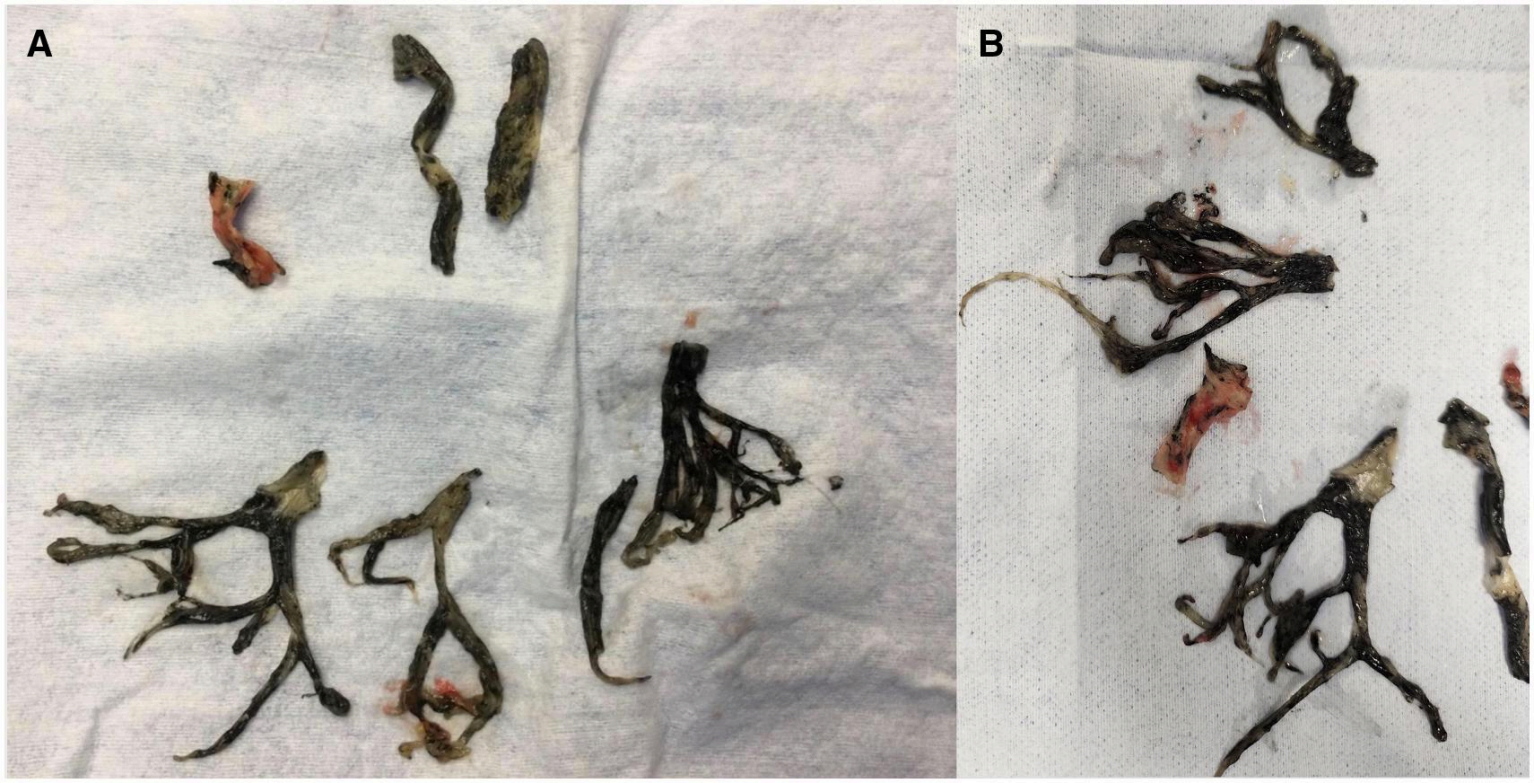
Mucosal pseudomembrane and slough tissue removed intra-operatively.

On day 3, ventilation was difficult with an elevated peak airway pressure of 50 cm H_2_O and a minimally effective tidal volume of 200 ml (3 ml/kg). Blood gas analysis revealed decompensated hypercapnic respiratory failure with pH 7.17 and a partial pressure of arterial carbon dioxide (PaCO_2_) of 14.9 kPa. The PaO_2_/FiO_2_ ratio was 93 at an FiO_2_ of 1.0 and a positive end-expiratory pressure of 10 cm H_2_O with the patient under full sedation and paralytic infusion. Fiberoptic bronchoscopy revealed extensive mucosal sloughing and pseudomembrane formation, causing endoluminal obstruction starting at the main tracheal level (Fig. 1A). Repeated attempts to remove the obstructive pseudomembrane with bronchoscopy at the bedside were unsuccessful (Fig. 1B and C). Veno-venous extracorporeal membrane oxygenation (VV-ECMO) was established to stabilize the patient and for periprocedural support. Pseudomembrane and slough removal with biopsy forceps was performed in the operating theatre by cardiothoracic surgeons with the patient under VV-ECMO support (Fig. 2A and B; Supplementary Video 1). The underlying inflamed mucosa was observed after a successful operation. Improvement in minute ventilation was evident immediately to 550 ml (8.5 ml/kg) after the operation. The patient was also tracheostomized because of significant upper airway oedema. The patient was successfully weaned off VV-ECMO support 1 day after the intrabronchial slough was removed. At the follow-up examination 1 month after hospital discharge, the patient was decannulated from the tracheostomy and remained well.

## DISCUSSION

Smoke exposure causes inhalation injury, which comprises thermal and chemical irritation of the upper airway and tracheobronchial system and delayed damage to the lung parenchyma [1]. The spectrum of tracheobronchial inhalation injuries ranges from mild mucosal erythema and carbonaceous deposits to mucosal sloughing and necrosis. The Abbreviated Injury Scale was developed to categorize patients according to the severity of the bronchoscopic findings from grades 0 to 4. It has been shown to correlate with the risk of progression to severe acute respiratory distress syndrome (ARDS) and ventilator dependence [2]. The pathophysiology of acute lung injury from smoke inhalation is multifactorial. Chemical irritation inhibits surfactant production, causing alveolar collapse and activating the release of reactive oxygen species into the bronchial circulation, which impairs hypoxic pulmonary vasoconstriction, resulting in ventilation–perfusion mismatch and severe ARDS [1, 3].

Veno-venous extracorporeal membrane oxygenation (VV-ECMO) is an effective rescue therapy for ARDS in which conventional ventilatory strategies fail. Its application in patients with burn injuries and ARDS was first described in the paediatric population [4] and was later extended to the adult population. In most reported studies, lung parenchymal injury from inhalation predominantly caused acute hypoxemic respiratory failure, requiring the initiation of VV-ECMO [5]. The formation of obstructive casts in the proximal airways, impeding ventilation and warranting VV-ECMO support, as in our case, has seldom been reported.

It is important to consider massive inhalation injury with mucosal sloughing and pseudomembrane formation, causing mechanical endoluminal obstruction when extreme difficulty in ventilation is encountered. Patients would benefit from frequent bronchoscopic evaluation during the first few days after smoke inhalation. Intrabronchial slough may form rapidly and can cause sudden deterioration as in our patient. Immediate bronchoscopic re-evaluation in case of any clinical deterioration is of paramount importance in prompt diagnosis and planning of interventions. One should consider VV-ECMO as a rescue therapy if conventional ventilatory strategies fail.

Traditionally, slough removal performed via rigid bronchoscopy would be technically easier and more straightforward. However, with the concern of significant laryngeal oedema and the possibility of losing the airway in this particular patient, slough removal was performed via flexible bronchoscopy instead, which is technically more demanding. The surgical technique required for removal of slough via flexible bronchoscopy can be seen in our supplementary video.

## Supplementary Material

ivad206_Supplementary_Data

## Data Availability

Data will be shared on reasonable request to the corresponding author.
